# Crystal structures and comparisons of huntite aluminum borates *RE*Al_3_(BO_3_)_4_ (*RE* = Tb, Dy and Ho)

**DOI:** 10.1107/S2056989020001802

**Published:** 2020-02-14

**Authors:** Saehwa Chong, Brian J. Riley, Zayne J. Nelson, Samuel N. Perry

**Affiliations:** a Pacific Northwest National Laboratory, Richland, WA 99354, USA; b Department of Civil and Environmental Engineering and Earth Sciences, University of Notre Dame, Notre Dame, IN 46556, USA

**Keywords:** huntite borate, lanthanide aluminum borate, single-crystal XRD

## Abstract

Three huntite-type aluminoborates of stoichiometry *RE*Al_3_(BO_3_)_4_ (*RE* = Tb, Dy and Ho), namely, terbium/dysprosium/holmium trialuminium tetrakis(borate), were synthesized by slow cooling within a K_2_Mo_3_O_10_ flux with spontaneous crystallization. The crystal structures were determined using single-crystal X-ray diffraction (SC-XRD) data. The synthesized borates are isostructural to the huntite [CaMg_3_(CO_3_)_4_] structure and crystallized within the trigonal *R*32 space group. The structural parameters were compared to literature data of other huntite *RE*Al_3_(BO_3_)_4_ crystals within the *R*32 space group. All three borates fit well into the trends calculated from the literature data. The unit-cell parameters and volumes increase linearly with larger *RE* cations whereas the densities decrease. All of the crystals studied were refined as inversion twins.

## Abstract 2

Three huntite-type aluminoborates of stoichiometry *RE*Al_3_(BO_3_)_4_ (*RE* = Tb, Dy and Ho) were synthesized by slow cooling within a K_2_Mo_3_O_10_ flux with spontaneous crystallization. The synthesized borates are isostructural to the huntite [CaMg(CO_3_)_4_] structure and crystallized within the trigonal *R*32 space group.

## Chemical context   

Rare-earth aluminum borates (REAB) with the general chemical formula *RE*Al_3_(BO_3_)_4_ (*RE* = La, Pr, Nd, Sm, Eu, Gd, Tb, Dy, Ho, Er, Tm, Yb, Lu, Y) have been studied extensively for applications in lasers, nonlinear optics, sensors, and phosphors because of their optical and magnetoelectric properties as well as the capacity to be doped with other rare-earth metals (Koporulina *et al.*, 2000 ▸; Leonyuk & Leonyuk, 1995 ▸; Leonyuk *et al.*, 1998 ▸; Mills, 1962 ▸; Belokoneva & Timchenko, 1983 ▸; Belokoneva, 1994 ▸). The REAB crystals are promising materials for self-frequency-doubling lasers as their nonlinear optical properties can be changed by doping with different rare-earth elements including Nd, Dy, Er, Yb, Tm, or Y (Leonyuk *et al.*, 1998 ▸, 2007 ▸; Földvári *et al.*, 2003 ▸; Chen *et al.*, 2012 ▸). The REAB compounds with *RE* = Tb, Ho, Er, or Tm exhibit the magnetoelectric properties useful for sensor applications (Liang *et al.*, 2011 ▸, 2012 ▸), and REAB with the *RE* = Pr, Sm, Eu, Gd, Tb, or Ho can be used as phosphors (Li & Wang, 2007 ▸; He *et al.*, 2015 ▸).

The REAB compounds are generally synthesized by a flux-assisted growth method with or without seeds at 800–1150°C (Leonyuk & Leonyuk, 1995 ▸; Koporulina *et al.*, 2000 ▸; Wang, 2012 ▸; Leonyuk, 2017 ▸). The K_2_Mo_3_O_10_ (Tu *et al.*, 1994 ▸; Wang *et al.*, 1995 ▸; Leonyuk & Leonyuk, 1995 ▸; Teshima *et al.*, 2006 ▸) compound is the most commonly used flux for the crystallization of REAB, although other fluxes such as Bi_2_O_3_–B_2_O_3_ (Chani *et al.*, 1994 ▸) and BaO–B_2_O_3_ (Jung *et al.*, 1995 ▸) have been used. Two major drawbacks of using the K_2_Mo_3_O_10_ flux are the potential incorporation of Mo into the REAB structure and co-crystallization of other phases (Wang, 2012 ▸; Leonyuk, 2017 ▸; Kuz’micheva *et al.*, 2019 ▸). In the current study, K_2_Mo_3_O_10_ flux was used to synthesize *RE*Al_3_(BO_3_)_4_ (*RE* = Tb, Dy, Ho) crystals, and the structural parameters of the synthesized REAB crystals were compared to literature data.

## Structural commentary   

**Figure 1:**
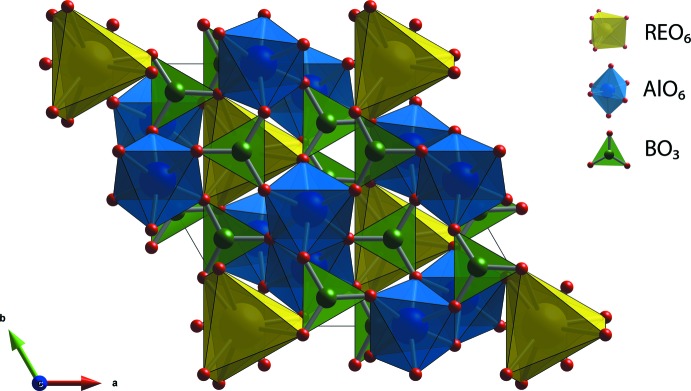
Crystal structure of *RE*Al_3_(BO_3_)_4_.

**Figure 2:**
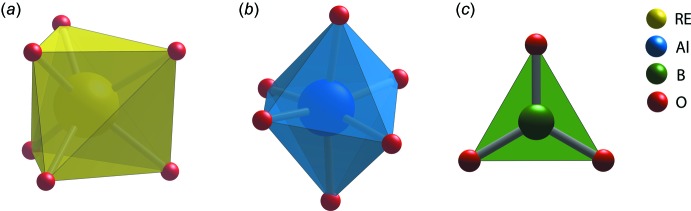
Coordination of oxygen atoms around (*a*) rare-earth, (*b*) aluminum, and (*c*) boron atoms shown as polyhedra.

**Figure 3:**
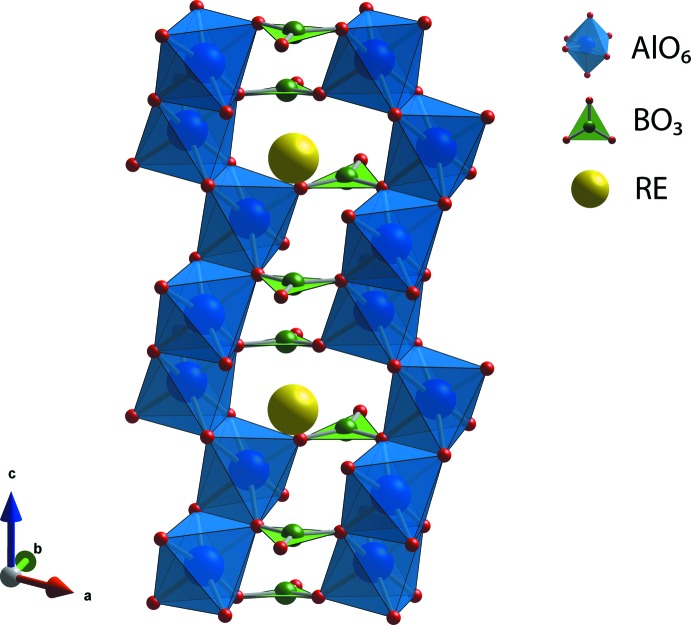
Structure showing the helical chains composed of edge sharing AlO_6_ units along the *c* axis connected by BO_3_ in *RE*Al_3_(BO_3_)_4_.

The crystal structures of the synthesized REAB crystals are isostructural to the huntite structure (Mills, 1962 ▸) with the *R*32 space group (Fig. 1 ▸). The huntite aluminoborates generally crystallize within the *R*32 space group; however, REAB compounds with *RE* = Pr, Nd, Sm, Eu, Tb, Ho, or Gd showed the transition in space group from *R*32 to lower symmetry monoclinic *C*2/*c* and *C*2 space groups in the disordered structures caused by variations in the growth temperature, cooling rate, and composition (Belokoneva & Timchenko, 1983 ▸; Belokoneva *et al.*, 1988 ▸, 1994 ▸; Leonyuk & Leonyuk, 1995 ▸; Plachinda & Belokoneva, 2008 ▸; Leonyuk, 2017 ▸). The structures of the REAB crystals are composed of rare-earth cations with a distorted trigonal–prismatic coordination (*RE*O_6_), aluminum cations with a distorted octa­hedral coord­ination (AlO_6_), and boron cations with a trigonal–planar coordination (BO_3_) as shown in Fig. 2 ▸. The AlO_6_ octa­hedra form helical chains along the *c*-axis direction by sharing edges, and these chains are connected by BO_3_ units (see Fig. 3 ▸).

**Figure 4:**
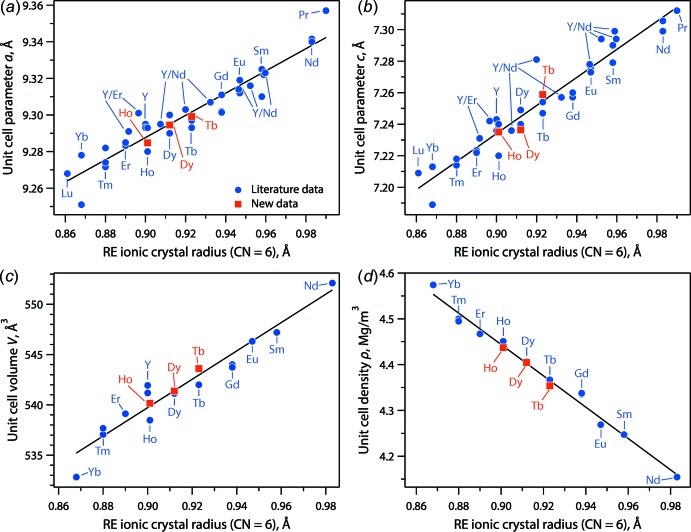
Summary of (*a*) unit-cell parameter *a*, (*b*) unit-cell parameter *c*, (*c*) unit-cell volume (*V*), and (*d*) density (ρ) as a function of the average ionic crystal radii of the *RE* in the crystal structures (coordination number = 6) from Shannon (1976 ▸).

The structural parameters of the synthesized REAB compounds were added to literature data for comparison (see Fig. 4 ▸), and they were in good agreement when plotted *versus* the average ionic crystal radius of the six-coordinated RE element according to Shannon (1976 ▸). The trendlines show that the unit-cell parameters and volumes increase linearly whereas the densities decrease with the larger rare-earth cations in the structures. The data included in Fig. 4 ▸ include literature data for *RE*Al_3_(BO_3_)_4_ where *RE* = Pr, Nd, Sm, Eu, Gd, Tb, Dy, Ho, Er, Tm, Yb, or Lu, as well as mixtures of Y/Er and Y/Nd (Belokoneva *et al.*, 1981 ▸; Hong & Dwight, 1974 ▸; Xu *et al.*, 2002 ▸; Jia *et al.*, 2006 ▸; Kuroda *et al.*, 1981 ▸; Leonyuk & Leonyuk, 1995 ▸; Malakhovskii *et al.*, 2014 ▸; Mészáros *et al.*, 2000 ▸; Mills, 1962 ▸; Plachinda & Belokoneva, 2008 ▸; Prokhorov *et al.*, 2013 ▸, 2014 ▸; Sváb *et al.*, 2012 ▸; Wang *et al.*, 1991 ▸).

## Synthesis and crystallization   

The REAB single crystals were synthesized using Tb_2_O_3_ (Alfa Aesar, 99.9%), Dy_2_O_3_ (Alfa Aesar, 99.9%), Ho_2_O_3_ (Alfa Aesar, 99.9%), Al(OH)_3_ (Almatis, 99.5%), B_2_O_3_ (Alfa Aesar, 99.98%), and K_2_Mo_3_O_10_ flux. All the rare-earth oxides, Al(OH)_3_, and B_2_O_3_ were used as received; the B_2_O_3_ was stored and handled in a nitro­gen glovebox to prevent hydration (M-Braun, Inc., < 0.1 ppm of O_2_ and H_2_O). The K_2_Mo_3_O_10_ flux was synthesized using K_2_CO_3_ (Alfa Aesar, 99%) and MoO_3_ (Alfa Aesar, 99.5%). For the flux, appropriate amounts of K_2_CO_3_ and MoO_3_ were mixed in a mortar and pestle and placed into a Pt/10%Rh crucible. The crucible was heated to 520°C at 5°C min^−1^, maintained at that temperature for 8 h, and then cooled down to room temperature at 5°C min^−1^. For the synthesis of the REAB crystals, the rare-earth oxide was mixed with Al(OH)_3_ and B_2_O_3_ in a 1:6:5 molar ratio, and then K_2_Mo_3_O_10_ was added at 40 mass% of the total precursor mass. The mixed powder of each rare-earth element was put into a Pt/10%Rh crucible, tightly covered with a Pt/10%Rh lid, and placed in a Thermolyne box furnace. The furnace was heated to 900°C at 5°C min^−1^, maintained at that temperature for 4 h, cooled to 400°C at 5°C h^−1^, and then shut off to cool naturally. The synthesized products were washed with deionized water in a sonic bath, and the crystals were recovered with vacuum filtration using a Büchner funnel. The REAB crystals along with KRE(MoO_4_)_2_ were synthesized by this process as expected from previous studies (Leonyuk *et al.*, 1998 ▸; Teshima *et al.*, 2006 ▸; Leonyuk, 2017 ▸; Kuz’micheva *et al.*, 2019 ▸).

**Figure 5:**
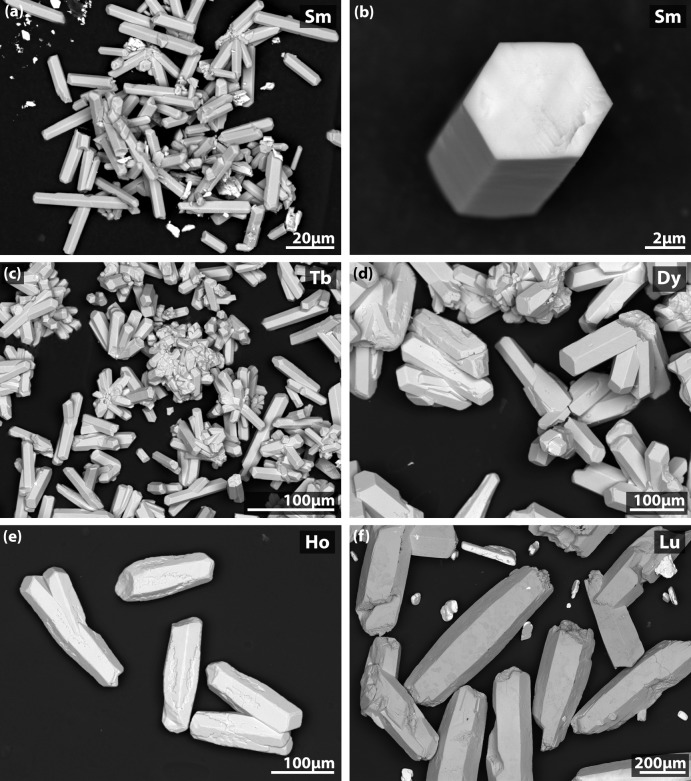
Back-scattered electron SEM micrographs of *RE*Al_3_(BO_3_)_4_ crystals including (*a*) and(*b*) SmAl_3_(BO_3_)_4_, (*c*) TbAl_3_(BO_3_)_4_, (*d*) DyAl_3_(BO_3_)_4_, (*e*) HoAl_3_(BO_3_)_4_, and (*f*) LuAl_3_(BO_3_)_4_. Note that some KLu(MoO_4_)_2_ crystals are seen in (*a*) and (*d*) as the smaller and brighter crystallites.

**Figure 6:**
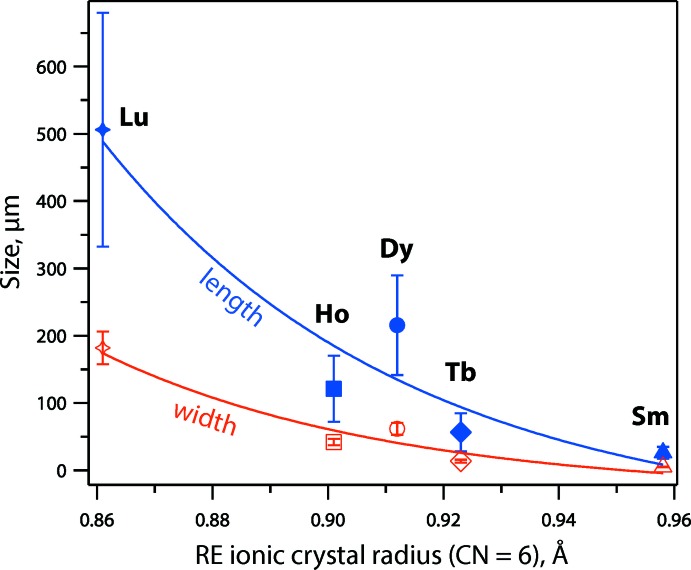
Summary of crystal size in terms of average length and width as a function of the average ionic crystal radii of the *RE* in the crystal structures (coordination number = 6) from Shannon (1976 ▸).

The REAB crystals generally have hexa­gonal prismatic shapes, and they were often agglomerated (Fig. 5 ▸), as observed with scanning electron microscopy (JSM-7001F field emission gun SEM; JEOL USA, Inc.). Crystals of SmAl_3_(BO_3_)_4_ and LuAl_3_(BO_3_)_4_ were also grown using the same procedure as described above and these are shown in Fig. 5 ▸ for comparison; however, the crystal structures are not reported due to the poor diffraction of SmAl_3_(BO_3_)_4_ and unresolvable displacement parameters during structural refinement for LuAl_3_(BO_3_)_4_. Finally, for the crystal growth conditions used here, the average crystallite sizes for the different REAl_3_(BO_3_)_4_ crystals herein (*RE* = Sm, Tb, Dy, Ho, Lu) are shown in Fig. 6 ▸ with standard deviations based on measurements of ≥ 7 crystals from each sample.

## Refinement   

Crystal data, data collection and structure refinement details are summarized in Table 1 ▸. Suitable crystals were selected for SC-XRD and were placed on cryoloops in oil (Parabar 10312, Hampton Research). Data were collected with a scan width of 0.5° in φ and ω with a 10 sec dwell time per frame at 100 K. All the REAB crystals had chiral structures and were refined with inversion twinning. The final refinements for TbAl_3_(BO_3_)_4_, DyAl_3_(BO_3_)_4_, and HoAl_3_(BO_3_)_4_ converged at *R*_1_ = 1.97% with goodness-of-fit of 1.08, *R*_1_ = 0.80% with goodness-of-fit of 1.14, and *R*_1_ = 1.15% with goodness-of-fit of 1.08, respectively.

## Supplementary Material

Crystal structure: contains datablock(s) global, Tb-borate, Dy-borate, Ho-borate. DOI: 10.1107/S2056989020001802/ru2069sup1.cif

Structure factors: contains datablock(s) Tb-borate. DOI: 10.1107/S2056989020001802/ru2069Tb-boratesup2.hkl

Structure factors: contains datablock(s) Dy-borate. DOI: 10.1107/S2056989020001802/ru2069Dy-boratesup3.hkl

Structure factors: contains datablock(s) Ho-borate. DOI: 10.1107/S2056989020001802/ru2069Ho-boratesup4.hkl

CCDC references: 1983041, 1983040, 1983039

Additional supporting information:  crystallographic information; 3D view; checkCIF report

## Figures and Tables

**Table 1:** Experimental details

	Tb-borate	Dy-borate	Ho-borate
Crystal data			
Chemical formula	TbAl_3_(BO_3_)_4_	DyAl_3_(BO_3_)_4_	HoAl_3_(BO_3_)_4_
*M*_r_	475.1	478.7	481.1
Crystal system, space group	Trigonal, *R*32	Trigonal, *R*32	Trigonal, *R*32
Temperature (K)	100	100	100
*a*, *c* (Å)	9.2992 (8), 7.2588 (7)	9.2938 (5), 7.2348 (4)	9.2832 (3), 7.2345 (3)
*V* (Å^3^)	543.61 (8)	541.18 (5)	539.93 (3)
*Z*	3	3	3
Radiation type	Mo *K*α	Mo *K*α	Mo *K*α
μ (mm^−1^)	10.21	10.81	11.45
Crystal size (mm)	0.03 × 0.03 × 0.02	0.05 × 0.05 × 0.03	0.05 × 0.05 × 0.03

Data collection			
Diffractometer	Bruker D8 QUEST CMOS area detector	Bruker D8 QUEST CMOS area detector	Bruker D8 QUEST CMOS area detector
Absorption correction	Multi-scan (*SADABS*; Krause *et al.*, 2015 ▸)	Multi-scan (*SADABS*; Krause *et al.*, 2015 ▸)	Multi-scan (*SADABS*; Krause *et al.*, 2015 ▸)
*T*_min_, *T*_max_	0.530, 0.747	0.588, 0.723	0.570, 0.709
No. of measured, independent and observed [*I* > 2σ(*I*)] reflections	6957, 270, 270	7295, 420, 420	5662, 381, 381
*R*_int_	0.113	0.044	0.047

Refinement			
*R*[*F* > 3σ(*F*)], *wR*(*F*), *S*	0.020, 0.021, 1.08	0.010, 0.010, 1.14	0.012, 0.015, 1.08
No. of reflections	270	420	381
No. of parameters	32	35	35
Δρ_max_, Δρ_min_ (e Å^−3^)	0.59, −0.88	0.29, −0.39	0.54, −0.65
Absolute structure	Refined as an inversion twin with a twin ratio of 0.51 (2):0.49 (2)	Refined as an inversion twin with a twin ratio of 0.509 (8):0.491 (8)	Refined as an inversion twin with a twin ratio of 0.558 (12):0.442 (12)
Absolute structure parameter	0.49 (2)	0.491 (8)	0.442 (12)

Computer programs: *APEX3* and *SAINT* (Bruker, 2012 ▸), *JANA2006* (Petříček *et al.*, 2014 ▸), *SUPERFLIP* (Palatinus & Chapuis, 2007 ▸), *VESTA* (Momma & Izumi, 2011 ▸) and *publCIF* (Westrip, 2010 ▸).

## supplementary crystallographic information

### Terbium trialuminium tetrakis(borate) (Tb-borate) Crystal data

**Table:**

TbAl_3_(BO_3_)_4_	*D*_x_ = 4.354 Mg m^−^^3^
*M**_r_* = 475.1	Mo *K*α radiation, λ = 0.71075 Å
Trigonal, *R*32	Cell parameters from 6957 reflections
Hall symbol: R 3 2"	θ = 3.8–33.2°
*a* = 9.2992 (8) Å	µ = 10.21 mm^−^^1^
*c* = 7.2588 (7) Å	*T* = 100 K
*V* = 543.61 (8) Å^3^	Hexagonal prism, light white
*Z* = 3	0.03 × 0.03 × 0.02 mm
*F*(000) = 660	

### Terbium trialuminium tetrakis(borate) (Tb-borate) Data collection

**Table:**

Bruker D8 QUEST CMOS area detector diffractometer	270 reflections with *I* > 2σ(*I*)
Radiation source: X-ray tube	*R*_int_ = 0.113
φ and ω scans	θ_max_ = 33.2°, θ_min_ = 3.8°
Absorption correction: multi-scan (SADABS; Krause *et al.*, 2015)	*h* = −13→14
*T*_min_ = 0.530, *T*_max_ = 0.747	*k* = −14→13
6957 measured reflections	*l* = −11→11
270 independent reflections	

### Terbium trialuminium tetrakis(borate) (Tb-borate) Refinement

**Table:**

Refinement on *F*	Primary atom site location: iterative
*R*[*F* > 3σ(*F*)] = 0.020	Weighting scheme based on measured s.u.'s *w* = 1/(σ^2^(*F*) + 0.0001*F*^2^)
*wR*(*F*) = 0.021	(Δ/σ)_max_ = 0.041
*S* = 1.08	Δρ_max_ = 0.59 e Å^−^^3^
270 reflections	Δρ_min_ = −0.88 e Å^−^^3^
32 parameters	Absolute structure: Data was refined with inversion twinning in JANA2006, and the twin ratio was 0.51(2):0.49(2).
0 restraints	Absolute structure parameter: 0.49 (2)
0 constraints	

### Terbium trialuminium tetrakis(borate) (Tb-borate) Special details

**Table:**

Refinement. Data was refined with inversion twinning, and the twin ratio was 0.51 (2):0.49 (2).

### Terbium trialuminium tetrakis(borate) (Tb-borate) Fractional atomic coordinates and isotropic or equivalent isotropic displacement parameters (Å^2^)

**Table:**

	*x*	*y*	*z*	*U*_iso_*/*U*_eq_	
Tb1	0	0	0	0.01126 (13)	
Al1	0.5557 (2)	0	0	0.0052 (6)	
B1	0.666667	0.333333	−0.166667	0.0054 (19)	
O1	0.7418 (7)	0.0752 (7)	0.166667	0.012 (2)	
O2	0.3668 (5)	−0.1151 (5)	−0.1448 (5)	0.0098 (12)	
O3	0.8151 (5)	0.4818 (5)	−0.166667	0.0091 (14)	
B2	0.8912 (11)	0.2245 (11)	0.166667	0.0066 (12)*	

### Terbium trialuminium tetrakis(borate) (Tb-borate) Atomic displacement parameters (Å^2^)

**Table:**

	*U*^11^	*U*^22^	*U*^33^	*U*^12^	*U*^13^	*U*^23^
Tb1	0.01027 (16)	0.01027 (16)	0.0133 (2)	0.00513 (8)	0	0
Al1	0.0040 (6)	0.0036 (8)	0.0078 (9)	0.0018 (4)	0.0005 (3)	0.0010 (6)
B1	0.004 (2)	0.004 (2)	0.008 (3)	0.0021 (12)	0	0
O1	0.007 (2)	0.007 (2)	0.017 (3)	0.000 (2)	0.0008 (9)	−0.0008 (9)
O2	0.0082 (16)	0.0086 (15)	0.0123 (15)	0.0039 (13)	0.0003 (12)	−0.0013 (12)
O3	0.0075 (15)	0.0075 (15)	0.011 (2)	0.0031 (18)	−0.0010 (8)	0.0010 (8)

### Terbium trialuminium tetrakis(borate) (Tb-borate) Geometric parameters (Å, º)

**Table:**

Tb1—O2^i^	2.336 (4)	B1—O3	1.381 (4)
Tb1—O2^ii^	2.336 (4)	B1—O3^vii^	1.381 (6)
Tb1—O2^iii^	2.336 (6)	B1—O3^viii^	1.381 (6)
Tb1—O2^iv^	2.336 (4)	O1—B2	1.389 (8)
Tb1—O2^v^	2.336 (4)	O2—B2^ix^	1.362 (12)
Tb1—O2^vi^	2.336 (6)		
			
O2^i^—Tb1—O2^ii^	89.15 (16)	O2^iii^—Tb1—O2^vi^	144.93 (12)
O2^i^—Tb1—O2^iii^	89.15 (16)	O2^iv^—Tb1—O2^v^	89.15 (16)
O2^i^—Tb1—O2^iv^	119.88 (12)	O2^iv^—Tb1—O2^vi^	89.15 (16)
O2^i^—Tb1—O2^v^	144.93 (19)	O2^v^—Tb1—O2^vi^	89.15 (16)
O2^i^—Tb1—O2^vi^	73.32 (15)	O3—B1—O3^vii^	120.0 (4)
O2^ii^—Tb1—O2^iii^	89.15 (16)	O3—B1—O3^viii^	120.0 (4)
O2^ii^—Tb1—O2^iv^	144.93 (19)	O3^vii^—B1—O3^viii^	120.0 (4)
O2^ii^—Tb1—O2^v^	73.32 (14)	Tb1^x^—O2—B2^ix^	105.0 (3)
O2^ii^—Tb1—O2^vi^	119.88 (18)	O1—B2—O2^xi^	117.7 (9)
O2^iii^—Tb1—O2^iv^	73.32 (15)	O1—B2—O2^xii^	117.7 (9)
O2^iii^—Tb1—O2^v^	119.88 (18)	O2^xi^—B2—O2^xii^	124.7 (6)

Symmetry codes: (i) *x*−1/3, *y*+1/3, *z*+1/3; (ii) −*y*−1/3, *x*−*y*−2/3, *z*+1/3; (iii) −*x*+*y*+2/3, −*x*+1/3, *z*+1/3; (iv) *y*+1/3, *x*−1/3, −*z*−1/3; (v) *x*−*y*−2/3, −*y*−1/3, −*z*−1/3; (vi) −*x*+1/3, −*x*+*y*+2/3, −*z*−1/3; (vii) −*y*+1, *x*−*y*, *z*; (viii) −*x*+*y*+1, −*x*+1, *z*; (ix) *x*−2/3, *y*−1/3, *z*−1/3; (x) *x*+1/3, *y*−1/3, *z*−1/3; (xi) *x*+2/3, *y*+1/3, *z*+1/3; (xii) *y*+1, *x*, −*z*.

### Dysprosium trialuminium tetrakis(borate) (Dy-borate) Crystal data

**Table:**

DyAl_3_(BO_3_)_4_	*D*_x_ = 4.406 Mg m^−^^3^
*M**_r_* = 478.7	Mo *K*α radiation, λ = 0.71075 Å
Trigonal, *R*32	Cell parameters from 7295 reflections
Hall symbol: R 3 2"	θ = 3.8–31.5°
*a* = 9.2938 (5) Å	µ = 10.81 mm^−^^1^
*c* = 7.2348 (4) Å	*T* = 100 K
*V* = 541.18 (5) Å^3^	Hexagonal prism, light white
*Z* = 3	0.05 × 0.05 × 0.03 mm
*F*(000) = 663	

### Dysprosium trialuminium tetrakis(borate) (Dy-borate) Data collection

**Table:**

Bruker D8 QUEST CMOS area detector diffractometer	420 reflections with *I* > 2σ(*I*)
Radiation source: X-ray tube	*R*_int_ = 0.044
φ and ω scans	θ_max_ = 31.5°, θ_min_ = 3.8°
Absorption correction: multi-scan (SADABS; Krause *et al.*, 2015)	*h* = −12→13
*T*_min_ = 0.588, *T*_max_ = 0.723	*k* = −13→13
7295 measured reflections	*l* = −10→10
420 independent reflections	

### Dysprosium trialuminium tetrakis(borate) (Dy-borate) Refinement

**Table:**

Refinement on *F*	Primary atom site location: iterative
*R*[*F* > 3σ(*F*)] = 0.010	Weighting scheme based on measured s.u.'s *w* = 1/(σ^2^(*F*) + 0.0001*F*^2^)
*wR*(*F*) = 0.010	(Δ/σ)_max_ = 0.017
*S* = 1.14	Δρ_max_ = 0.29 e Å^−^^3^
420 reflections	Δρ_min_ = −0.39 e Å^−^^3^
35 parameters	Absolute structure: The crystal had chiral structure. Data was refined with inversion twinning in JANA2006, and the twin ratio was 0.509(8):0.491(8).
0 restraints	Absolute structure parameter: 0.491 (8)
0 constraints	

### Dysprosium trialuminium tetrakis(borate) (Dy-borate) Special details

**Table:**

Refinement. Data was refined with inversion twinning, and the twin ratio was 0.509 (8):0.491 (8).

### Dysprosium trialuminium tetrakis(borate) (Dy-borate) Fractional atomic coordinates and isotropic or equivalent isotropic displacement parameters (Å^2^)

**Table:**

	*x*	*y*	*z*	*U*_iso_*/*U*_eq_	
Dy1	0	0	0	0.00585 (4)	
Al1	0.55590 (10)	0	0	0.0031 (2)	
O1	0.7422 (3)	0.0755 (3)	0.166667	0.0084 (9)	
B1	0.666667	0.333333	−0.166667	0.0062 (7)	
O3	0.36691 (18)	−0.11579 (17)	−0.14502 (18)	0.0065 (4)	
O2	0.8159 (2)	0.4825 (2)	−0.166667	0.0067 (5)	
B2	0.8916 (5)	0.2249 (5)	0.166667	0.0063 (10)	

### Dysprosium trialuminium tetrakis(borate) (Dy-borate) Atomic displacement parameters (Å^2^)

**Table:**

	*U*^11^	*U*^22^	*U*^33^	*U*^12^	*U*^13^	*U*^23^
Dy1	0.00546 (6)	0.00546 (6)	0.00665 (6)	0.00273 (3)	0	0
Al1	0.0027 (2)	0.0023 (3)	0.0042 (3)	0.00114 (15)	−0.00007 (10)	−0.0001 (2)
O1	0.0075 (11)	0.0075 (11)	0.0082 (10)	0.0022 (11)	−0.0006 (4)	0.0006 (4)
B1	0.0068 (9)	0.0068 (9)	0.0051 (13)	0.0034 (4)	0	0
O3	0.0058 (6)	0.0063 (5)	0.0072 (5)	0.0029 (4)	−0.0011 (4)	−0.0013 (4)
O2	0.0060 (6)	0.0060 (6)	0.0071 (7)	0.0022 (7)	−0.0004 (3)	0.0004 (3)
B2	0.0083 (11)	0.0083 (11)	0.0058 (9)	0.0068 (15)	−0.0001 (5)	0.0001 (5)

### Dysprosium trialuminium tetrakis(borate) (Dy-borate) Geometric parameters (Å, º)

**Table:**

Dy1—O3^i^	2.3260 (16)	Al1—Al1^viii^	2.9992 (7)
Dy1—O3^ii^	2.3260 (13)	O1—B2	1.388 (4)
Dy1—O3^iii^	2.326 (2)	B1—O2	1.3866 (13)
Dy1—O3^iv^	2.3260 (16)	B1—O2^ix^	1.387 (2)
Dy1—O3^v^	2.3260 (13)	B1—O2^x^	1.387 (2)
Dy1—O3^vi^	2.326 (2)	O3—B2^xi^	1.364 (6)
Al1—Al1^vii^	2.9992 (7)		
			
O3^i^—Dy1—O3^ii^	89.16 (6)	O3^iv^—Dy1—O3^v^	89.16 (6)
O3^i^—Dy1—O3^iii^	89.16 (6)	O3^iv^—Dy1—O3^vi^	89.16 (6)
O3^i^—Dy1—O3^iv^	119.78 (5)	O3^v^—Dy1—O3^vi^	89.16 (6)
O3^i^—Dy1—O3^v^	145.03 (7)	Al1^vii^—Al1—Al1^viii^	118.02 (3)
O3^i^—Dy1—O3^vi^	73.33 (6)	O2—B1—O2^ix^	120.00 (13)
O3^ii^—Dy1—O3^iii^	89.16 (6)	O2—B1—O2^x^	120.00 (13)
O3^ii^—Dy1—O3^iv^	145.03 (7)	O2^ix^—B1—O2^x^	120.00 (13)
O3^ii^—Dy1—O3^v^	73.33 (5)	Dy1^xii^—O3—B2^xi^	105.29 (15)
O3^ii^—Dy1—O3^vi^	119.78 (7)	O1—B2—O3^xiii^	117.3 (4)
O3^iii^—Dy1—O3^iv^	73.33 (6)	O1—B2—O3^xiv^	117.3 (4)
O3^iii^—Dy1—O3^v^	119.78 (7)	O3^xiii^—B2—O3^xiv^	125.4 (3)
O3^iii^—Dy1—O3^vi^	145.03 (5)		

Symmetry codes: (i) *x*−1/3, *y*+1/3, *z*+1/3; (ii) −*y*−1/3, *x*−*y*−2/3, *z*+1/3; (iii) −*x*+*y*+2/3, −*x*+1/3, *z*+1/3; (iv) *y*+1/3, *x*−1/3, −*z*−1/3; (v) *x*−*y*−2/3, −*y*−1/3, −*z*−1/3; (vi) −*x*+1/3, −*x*+*y*+2/3, −*z*−1/3; (vii) −*y*+2/3, *x*−*y*−2/3, *z*+1/3; (viii) −*x*+*y*+4/3, −*x*+2/3, *z*−1/3; (ix) −*y*+1, *x*−*y*, *z*; (x) −*x*+*y*+1, −*x*+1, *z*; (xi) *x*−2/3, *y*−1/3, *z*−1/3; (xii) *x*+1/3, *y*−1/3, *z*−1/3; (xiii) *x*+2/3, *y*+1/3, *z*+1/3; (xiv) *y*+1, *x*, −*z*.

### Holmium trialuminium tetrakis(borate) (Ho-borate) Crystal data

**Table:**

HoAl_3_(BO_3_)_4_	*D*_x_ = 4.439 Mg m^−^^3^
*M**_r_* = 481.1	Mo *K*α radiation, λ = 0.71075 Å
Trigonal, *R*32	Cell parameters from 5662 reflections
Hall symbol: R 3 2"	θ = 3.8–30.5°
*a* = 9.2832 (3) Å	µ = 11.45 mm^−^^1^
*c* = 7.2345 (3) Å	*T* = 100 K
*V* = 539.93 (3) Å^3^	Hexagonal prism, light pink
*Z* = 3	0.05 × 0.05 × 0.03 mm
*F*(000) = 666	

### Holmium trialuminium tetrakis(borate) (Ho-borate) Data collection

**Table:**

Bruker D8 QUEST CMOS area detector diffractometer	381 reflections with *I* > 2σ(*I*)
Radiation source: X-ray tube	*R*_int_ = 0.047
φ and ω scans	θ_max_ = 30.5°, θ_min_ = 3.8°
Absorption correction: multi-scan (SADABS; Krause *et al.*, 2015)	*h* = −13→13
*T*_min_ = 0.570, *T*_max_ = 0.709	*k* = −13→12
5662 measured reflections	*l* = −10→10
381 independent reflections	

### Holmium trialuminium tetrakis(borate) (Ho-borate) Refinement

**Table:**

Refinement on *F*	Primary atom site location: iterative
*R*[*F* > 3σ(*F*)] = 0.012	Weighting scheme based on measured s.u.'s *w* = 1/(σ^2^(*F*) + 0.0001*F*^2^)
*wR*(*F*) = 0.015	(Δ/σ)_max_ = 0.036
*S* = 1.08	Δρ_max_ = 0.54 e Å^−^^3^
381 reflections	Δρ_min_ = −0.65 e Å^−^^3^
35 parameters	Absolute structure: The crystal had chiral structure. Data was refined with inversion twinning in JANA2006, and the twin ratio was 0.558(12):0.442(12).
0 restraints	Absolute structure parameter: 0.442 (12)
0 constraints	

### Holmium trialuminium tetrakis(borate) (Ho-borate) Special details

**Table:**

Refinement. Data was refined with inversion twinning, and the twin ratio was 0.558 (12):0.442 (12).

### Holmium trialuminium tetrakis(borate) (Ho-borate) Fractional atomic coordinates and isotropic or equivalent isotropic displacement parameters (Å^2^)

**Table:**

	*x*	*y*	*z*	*U*_iso_*/*U*_eq_	
Ho1	0	0	0	0.01041 (7)	
Al1	0.55519 (15)	0	0	0.0062 (3)	
B1	0.666667	0.333333	−0.166667	0.0090 (12)	
O1	0.7428 (5)	0.0762 (5)	0.166667	0.0123 (14)	
O3	0.3668 (3)	−0.1161 (3)	−0.1461 (3)	0.0101 (7)	
O2	0.8155 (3)	0.4822 (3)	−0.166667	0.0094 (8)	
B2	0.8911 (8)	0.2245 (8)	0.166667	0.0105 (16)	

### Holmium trialuminium tetrakis(borate) (Ho-borate) Atomic displacement parameters (Å^2^)

**Table:**

	*U*^11^	*U*^22^	*U*^33^	*U*^12^	*U*^13^	*U*^23^
Ho1	0.00996 (9)	0.00996 (9)	0.01130 (10)	0.00498 (4)	0	0
Al1	0.0058 (4)	0.0056 (5)	0.0071 (5)	0.0028 (2)	−0.00023 (15)	−0.0005 (3)
B1	0.0105 (15)	0.0105 (15)	0.0060 (18)	0.0053 (8)	0	0
O1	0.0107 (17)	0.0107 (17)	0.0140 (16)	0.0044 (17)	−0.0021 (7)	0.0021 (7)
O3	0.0100 (10)	0.0098 (9)	0.0106 (7)	0.0050 (7)	−0.0004 (6)	−0.0009 (7)
O2	0.0078 (9)	0.0078 (9)	0.0119 (10)	0.0033 (11)	0.0000 (5)	0.0000 (5)
B2	0.0131 (18)	0.0131 (18)	0.0090 (15)	0.009 (2)	0.0006 (9)	−0.0006 (9)

### Holmium trialuminium tetrakis(borate) (Ho-borate) Geometric parameters (Å, º)

**Table:**

Ho1—O3^i^	2.318 (3)	B1—O2	1.382 (2)
Ho1—O3^ii^	2.318 (2)	B1—O2^vii^	1.382 (4)
Ho1—O3^iii^	2.318 (3)	B1—O2^viii^	1.382 (4)
Ho1—O3^iv^	2.318 (3)	O1—B2	1.377 (6)
Ho1—O3^v^	2.318 (2)	O3—B2^ix^	1.364 (9)
Ho1—O3^vi^	2.318 (3)		
			
O3^i^—Ho1—O3^ii^	89.28 (9)	O3^iii^—Ho1—O3^vi^	144.97 (7)
O3^i^—Ho1—O3^iii^	89.28 (9)	O3^iv^—Ho1—O3^v^	89.28 (9)
O3^i^—Ho1—O3^iv^	119.73 (7)	O3^iv^—Ho1—O3^vi^	89.28 (9)
O3^i^—Ho1—O3^v^	144.97 (12)	O3^v^—Ho1—O3^vi^	89.28 (9)
O3^i^—Ho1—O3^vi^	73.16 (9)	O2—B1—O2^vii^	120.0 (2)
O3^ii^—Ho1—O3^iii^	89.28 (9)	O2—B1—O2^viii^	120.0 (2)
O3^ii^—Ho1—O3^iv^	144.97 (12)	O2^vii^—B1—O2^viii^	120.0 (2)
O3^ii^—Ho1—O3^v^	73.16 (8)	Ho1^x^—O3—B2^ix^	105.5 (2)
O3^ii^—Ho1—O3^vi^	119.73 (11)	O1—B2—O3^xi^	117.4 (7)
O3^iii^—Ho1—O3^iv^	73.16 (9)	O1—B2—O3^xii^	117.4 (7)
O3^iii^—Ho1—O3^v^	119.73 (11)	O3^xi^—B2—O3^xii^	125.3 (4)

Symmetry codes: (i) *x*−1/3, *y*+1/3, *z*+1/3; (ii) −*y*−1/3, *x*−*y*−2/3, *z*+1/3; (iii) −*x*+*y*+2/3, −*x*+1/3, *z*+1/3; (iv) *y*+1/3, *x*−1/3, −*z*−1/3; (v) *x*−*y*−2/3, −*y*−1/3, −*z*−1/3; (vi) −*x*+1/3, −*x*+*y*+2/3, −*z*−1/3; (vii) −*y*+1, *x*−*y*, *z*; (viii) −*x*+*y*+1, −*x*+1, *z*; (ix) *x*−2/3, *y*−1/3, *z*−1/3; (x) *x*+1/3, *y*−1/3, *z*−1/3; (xi) *x*+2/3, *y*+1/3, *z*+1/3; (xii) *y*+1, *x*, −*z*.
